# Drivers for enhancing job performance of prison officers in Slovenia: effects of job attitudes, organizational, and work-related factors

**DOI:** 10.3389/fpsyg.2023.1247743

**Published:** 2023-09-08

**Authors:** Katrin Podgorski, Branko Lobnikar, Anže Mihelič, Kaja Prislan Mihelič

**Affiliations:** ^1^General Police Directorate, Ministry of the Interior, Ljubljana, Slovenia; ^2^Faculty of Criminal Justice and Security, University of Maribor, Ljubljana, Slovenia

**Keywords:** job performance, task performance, workplace psychology, prison, prison officer

## Abstract

Maintaining order and safety in a prison environment heavily depends on prison officers, who daily interact with prisoners and are constantly dealing with dangerous situations. Their task performance is vital for the organizational performance and the fulfillment of the prisons’ mission. For managing prison officers’ job performance efficiently, it is important to understand the associated factors; however, job performance in a prison environment remains completely unexplored in Slovenia. This article presents a study conducted among Slovenian prison officers (*n* = 201), which examined their task performance, its association with job attitudes, and the role of organizational and work-related factors. The study results showed that the prison officers’ task performance is associated with their job satisfaction, but not with their job involvement. Moreover, their job satisfaction is associated with perceived organizational justice, job stress, and the dangerousness of the job. Based on these findings, we demonstrated that task performance depends on several direct and indirect factors that prison management should prioritize, the key ones being stress reduction, strengthening the feeling of organizational justice, and the ability to deal with the job-related dangers successfully. This article highlights organizational and work-related factors important for improving the prison officers’ well-being at work.

## 1. Introduction

Employee job performance, which refers to positive employee behavior and the creation of expected outcomes of their work, has an important added value for organizations because it contributes to general organizational efficiency and effectiveness (Motowidlo et al., 1997; Jex and Britt, 2008; Kumari and Thapliyal, 2017). Proactive, involved, and high-performing employees are essential for achieving organizational objectives (Motowidlo and Kell, 2013); therefore, employee job performance management and evaluation should be part of any organization’s strategic management processes. Regular and consistent evaluation of job performance is important for several reasons: it gives the management insight into the quality of work, and, at the same time, it makes it possible to understand which organizational and work-related factors influence the employees’ work. Based on this, the methods of work can be adapted and potential improvements in employee job performance can be planned (Judge et al., 2001; Vandenabeele, 2009; Johari and Yahya, 2016; Thevanes and Dirojan, 2018).

Managing and evaluating job performance is also exceptionally important in prisons where, due to the nature of the work – involving employees with a high level of authority and powers – it is vital that work is carried out lawfully and professionally. In addition, such employees (e.g., police or prison officers) are often exposed to dangerous, stressful, and demanding situations that affect their job performance (Lambert et al., 2012; Frank et al., 2019). In line with the growing demands for higher efficiency of the law enforcement system and overpopulated prison institutions, prison officers are also often faced with an increased workload and are consequently overburdened. Hence, studies aimed at understanding what drives their performance are even more essential. Moreover, monitoring and promoting job performance and the high quality of work of prison officers are important since they are key actors in ensuring order, safety, and the legitimacy of the prison system as a whole (Meško et al., 2016; Meško and Hacin, 2019).

Exploring job performance is a challenge for researchers, because it is a complex concept composed of various dimensions (e.g., task performance, contextual performance, and organizational citizenship behavior; Borman and Motowidlo, 1997; Motowidlo et al., 1997; Organ, 1997). Even though research on employee job performance is fairly widespread within various organizational environments, it is generally less common and narrower in law enforcement and security organizations. For example, studies within the context of prisons and the police largely focus on specific aspects of job performance, such as organizational citizenship behavior (OCB) (e.g., Lambert, 2010; Lambert et al., 2012; Frank et al., 2019) or the counterproductive work behavior (CWB) of employees (e.g., Reisig and Mesko, 2009; Wolfe and Piquero, 2011; Boateng and Hsieh, 2019a), but they only rarely explore task performance, which is one of the most important dimensions of job performance (Lambert et al., 2012, 2014).

Another challenge is the understanding of factors associated with the prison officers’ job performance. Past research on job performance at prisons has focused on organizational and work-related factors, establishing the cause-effect relationships between them (Lambert et al., 2008, 2019; Paoline and Lambert, 2012; Qureshi et al., 2019) and only rarely exploring the association of job performance with other factors, such as job attitudes. Specifically, organizational research has already shown that job involvement and job satisfaction as important aspects of employee job attitudes are influential factors associated with employee job performance (Judge et al., 2001; Castle, 2008; Riketta, 2008; Thevanes and Dirojan, 2018), but they remain unexplored in studies of employees at law enforcement and security organizations.

In addition to the above, another challenge evident from the literature is the various methods of measuring job performance and the different organizational and cultural contexts of the studies. For example, job performance studies in prisons are usually conducted in different organizational settings (e.g., private prisons, high-security prisons, etc.) and countries with different concepts and methodologies. This leads to inconsistent findings, whose interpretation requires considering the research context (i.e., environment or situation).

In line with the challenges described above, researchers often draw attention to a general lack of consistency in studying the job performance of police officers (Shane, 2010; Frank et al., 2019) and prison officers (Lambert, 2010), highlighting the need for more detailed and systematic research into factors influencing the job performance of the latter (Lambert and Paoline, 2008; Lambert, 2010).

Similar challenges and deficiencies can also be observed in Slovenian research. Even though studies of the Slovenian prison system are relatively well-established and widespread, in the past, researchers have mainly focused on determining the employees’ and inmates’ views on the social climate and examining the legitimacy of the prison staff (Brinc, 2011; Meško et al., 2016; Meško and Hacin, 2019; Hacin and Meško, 2021). However, to date, no research has been conducted that focuses on job performance in the Slovenian prison system. Because past studies that have already explored job performance at prisons facilitated the development of more effective management approaches, such studies should also be promoted in the Slovenian prison environment.

The main objective of this article is thus to explore: (a) the degree of task performance of prison officers in Slovenia; (b) how their task performance is associated with their job attitudes, and (c) how these attitudes are associated with organizational and work-related factors.

## 2. Theoretical background

Job performance can be described as expected achievements and results of employees in an organization, which result from the long-term repetition of desired behavior and affect the achievement of organizational objectives (Motowidlo and Kell, 2013). Researchers describe job performance as a complex and multidimensional construct (Borman and Motowidlo, 1997; Griffin et al., 2000; Motowidlo and Kell, 2013). The two job performance dimensions most frequently mentioned in the literature are task performance and contextual performance (Motowidlo and Van Scotter, 1994; Motowidlo et al., 1997; Griffin et al., 2000). However, some authors divide job performance into three dimensions: task performance, contextual performance, and CWB (Rotundo and Sackett, 2002; Dalal, 2005).

Task performance refers to patterns of behavior that help achieve the expected results (Van Scotter et al., 2000); this behavior involves the fulfillment of the basic job requirements (Motowidlo et al., 1997; Rotundo and Sackett, 2002; Pradhan and Jena, 2017). It can also be defined as the performance of activities that usually appear on formal job descriptions and behavior that contributes to reaching the organization’s objectives through the performance of certain tasks (Motowidlo and Van Scotter, 1994; Borman and Motowidlo, 1997; Jex and Britt, 2008; Motowidlo and Kell, 2013). In previous research, task performance has been highlighted as one of the most important dimensions of employee job performance, because it refers explicitly to the fulfillment of the basic and formally defined job requirements (Rotundo and Sackett, 2002; Jex and Britt, 2008). Despite the recommended application of several dimensions in studying employee job performance, task performance is a sufficient and in practice, the most illustrative indicator for understanding employee performance and efficiency at work.

Moreover, contextual performance can be understood as a discretionary, voluntary employee behavior that does not depend on the reward systems in the organization, but contributes to its effective functioning and productivity and helps create a good organizational climate and culture (Organ, 1997; Pradhan and Jena, 2017). It includes aspects such as prosocial organizational behavior, organizational spontaneity, and OCB (Griffin et al., 2000). Contextual performance is thus associated with proactive behavior that is not required in the job description and is instead a matter of the employees’ personal choices. This type of performance also includes OCB (Motowidlo and Van Scotter, 1994; Borman and Motowidlo, 1997), which has an important impact on organizational effectiveness and employee productivity (Podsakoff et al., 2000). In the literature, the OCB concept is often divided into several aspects, for example: (1) altruism, conscientiousness, sportsmanship, courtesy, and civic virtue (Organ, 1997), (2) organizational citizenship behavior toward the organization (OCB-O) and organizational citizenship behavior toward individuals (OCB-I) (Williams and Anderson, 1991; Dalal, 2005), or (3) altruism and compliance (Lambert, 2010; Frank et al., 2019). Williams and Anderson (1991) suggested that the first five OCB dimensions could be combined into their two constructs: OCB-O and OCB-I. The OCB-I construct would include altruism and courtesy, and the OCB-O construct would comprise conscientiousness, civic virtue, and sportsmanship.

However, another important job performance dimension is CWB, which harms the organization and has a negative impact on organizational performance. This refers to voluntary behavior at work that harms the well-being of the organization or its members (Rotundo and Sackett, 2002). Motowidlo and Kell (2013) also refer to this concept or dimension as dysfunctional organizational behavior, and other researchers refer to it as employee misconduct (Boateng and Hsieh, 2019a).

From a literature review, it is evident that employee job performance studies are carried out at various organizations, such as manufacturing companies, financial, health, and educational institutions, as well as government and public organizations (Hermawati and Mas, 2017; Kumari and Thapliyal, 2017; Thevanes and Dirojan, 2018). Moreover, past research shows that job performance is the result of various organizational, personal, and work-related factors. Because of this, it must be assessed within the context of interrelated factors.

Job performance evaluations are especially important in law enforcement and security organizations, which are key to ensuring and maintaining social stability and safeguarding fundamental social values, functions, and rights. Due to their role in society and the nature of work, employees in these organizations must perform their duties lawfully and professionally. Employee job performance in law enforcement and security organizations is also important because the perceived legitimacy of the organization and its employees depends on the quality of employees’ work. All this also applies to prison staff, especially prison officers, who are the bearers of power and authority, and whose task performance has a strong impact on prison security and the fulfillment of the prisons’ fundamental mission (Meško et al., 2014). Failure to perform tasks, poor work performance, deviations from the formal job requirements, and CWB can have exceptionally wide implications for effective prison management. They can lead to the emergence of risks in prisons and negatively affect the purpose of a prison sentence and, ultimately, general organizational efficiency and effectiveness.

Another important feature of working in a prison environment, which can affect employees and thus must be taken into account when studying their job performance, is the fact that the employees carry out dangerous work and are often exposed to stressful situations (Meško et al., 2004; Reisig and Mesko, 2009). This can lead to various psychological states and consequently affect the prison officers’ quality of work, work engagement, and job involvement. Therefore, a high job performance, a strong sense of safety among employees, and their positive psychological states are key to fulfilling the prison systems’ vision, objectives, and mission.

Based on all the aforementioned specific circumstances of working in prisons, which are not typical of other organizational environments, it is vital to not only monitor employee job performance, but also understand the factors associated with it.

## 3. Related work

Various studies of prison performance can be found in the literature; however, the focus is often on establishing prison performance in general. This involves analyses of various performance indicators, including official prison statistics and inmate surveys, as well as comparisons between public and private prisons, and the evaluations of inmate recidivism as a measure of prison performance (Camp et al., 2002; Spivak and Sharp, 2008). Meanwhile, studies of employee job performance in law enforcement and the associated factors started to receive increased researchers’ attention only in the new millennium.

Literature on employee job performance in prisons most often focuses on exploring the various dimensions of OCB (Lambert et al., 2012, 2014) and CWB or employee misconduct (Boateng and Hsieh, 2019a). Overall, research shows that job performance at prisons is associated with various personal, organizational, and work-related factors, such as the approach to employee management (clarity of tasks, feedback, and organizational justice) (Lambert et al., 2012; Lambert and Hogan, 2013). Personal and demographic factors (e.g., race, gender, age, work experience, rank, and education) are also shown to play an important role (Shane, 2010; Lambert et al., 2014; Frank et al., 2019). However, in this regard, Hogan et al. (2009) highlight that organizational factors are more important performance indicators than personal characteristics (race, age, gender, etc.).

Some studies have also focused on establishing the contribution of job attitudes to job performance in prisons. The elements of job attitudes include well-being at work, organizational commitment, life satisfaction, job satisfaction, and job involvement (Lambert, 2010; Frank et al., 2019). Moreover, some studies show that job attitudes are further associated with factors such as organizational justice, job stress, and stressors (Lambert et al., 2012; Boateng and Hsieh, 2019b).

A few limitations emerge in line with the review of the existing body of literature. As already noted, studies exploring the influence and significance of factors associated with prison employees’ job performance vary significantly. This may be a consequence of several circumstances; different cultural, systemical, and organizational environments of the studies performed; different dimensions of job performance being the focus of the research; and different approaches to measuring the performance (employees’ self-reporting or supervisors’ evaluations).

Even though several studies have been conducted in the Slovenian prison environment, there is a lack of literature exploring job performance in this context. Specifically, no research has yet been conducted on the perceived or self-reported job performance of prison officers in the Slovenian prison environment. Indirectly, this topic is at present best covered in the literature by studies examining the social climate at Slovenian prisons. This topic began to be explored in 1980, after which studies were conducted every five years (Brinc, 2011). A detailed overview of penological research in Slovenia was provided by Hacin (2015), who established that not only has the field of research and importance of studying prison-related issues expanded over time, but the purposes of a prison sentence have also changed, shifting from a sentencing ideology to rehabilitation. Accordingly, awareness of the importance of adequate staff qualifications and good relations with inmates has also strengthened. In addition, research on the (perceived and self-perceived) legitimacy of prison officers and other staff at Slovenian prisons has also become well established (e.g., Reisig and Mesko, 2009; Meško et al., 2016; Meško and Hacin, 2019). In relation to the topic addressed in this article, a study of the importance of prison officers’ professional skills in performing their work (Meško et al., 2004) provided some interesting insights. This study showed that, according to the prison officers themselves, at least one year of work experience is required for them to be able to perform their job with competence. The prison officers included in this study also expressed a need for more training and improving their communication skills.

## 4. Research framework

Based on the literature review on prison officers’ job performance, we can highlight two evident research gaps, i.e., the lack of studies exploring the prison officers’ task performance and the lack of research on job performance in the Slovenian prison environment. Because job performance can be affected by various personal, organizational and work-related factors, this article aims to address the following research questions (RQ):

*RQ1*: What is the task performance of prison officers?

*RQ2*: Are prison officers’ job attitudes associated with their task performance?

*RQ3*: Which organizational and work-related factors are associated with prison officers’ job attitudes?

In line with these research questions, a theoretical research model was designed based on the assumption that task performance is associated with job attitudes and job attitudes are associated with organizational and workplace factors. These correlations were defined based on the literature review and the findings of previous studies conducted at police organizations and within the prison environment. The basic concepts relevant for our study, hypotheses and their premises are presented in more detail in the following paragraphs.

### 4.1. Job attitudes

Employees’ job attitudes are key to their job performance. Job satisfaction and job involvement have been among the more important dimensions of job attitudes identified in studies to date (Lambert, 2010; Hermawati and Mas, 2017; Frank et al., 2019). Job involvement can be defined as an individual’s cognitive and psychological identification with their work (Kanungo, 1982). Job satisfaction refers to the feeling reflecting the extent to which a person’s needs are met at work, and is a result of interactions between the individual and the workplace environment (Griffin et al., 2010).

The findings of a meta-analysis (Riketta, 2008) showed that employees’ job satisfaction is positively associated with their performance. Similarly, Frank et al. (2019) and Vandenabeele (2009) also established that employees who are more satisfied with their jobs are more willing to perform tasks not included in their official job requirements, while job dissatisfaction reduces their job performance. Job involvement can have a similar influence on job performance, because employees showing greater job involvement usually have higher performance or are more willing to respect the rules and regulations within the organization, and exhibit more altruistic behavior at work (Hermawati and Mas, 2017; Thevanes and Dirojan, 2018; Frank et al., 2019). However, it should be noted that some studies have not confirmed these correlations (Lambert et al., 2008). Based on this, the following hypotheses were formulated:

*H1a*: Job satisfaction is positively associated with prison officers’ task performance.

*H1b*: Job involvement is positively associated with prison officers’ task performance.

### 4.2. Organizational and work-related factors

Just like job performance, job satisfaction and job involvement are complex concepts influenced by many organizational and work-related factors. The most important among these are presented below.

Organizational justice or organizational fairness is a concept that is often associated with employee task performance (Wolfe and Piquero, 2011; Boateng and Hsieh, 2019a). It refers to the employees’ perception that their organization treats them and their co-workers fairly and with justice (Lambert et al., 2019). Even though it is composed of several dimensions, such as procedural and distributive justice (Boateng and Hsieh, 2019a), this concept is often evaluated comprehensively, with a single construct (e.g., Ambrose and Schminke, 2009; Lambert and Hogan, 2009). Research shows that perceived organizational justice is associated with job satisfaction (Ambrose and Schminke, 2009; Qureshi et al., 2017); if an organization treats its employees fairly – based on the effort they invest and with fair procedures – they will be more satisfied with their job. The influence of perceived organizational justice on job satisfaction among prison officers and other staff in a prison environment has been explored by Jiang et al. (2018), Lambert et al. (2018), and Boateng and Hsieh (2019b).

Job satisfaction is also heavily influenced by the dangerousness of the job, which is an integral part of any police and prison officer’s job. Some authors (e.g., Cullen et al., 1985) define dangerousness of the job as perceptions of feeling at risk of injury at work, which prison staff can be very frequently exposed to due to the nature of their work with convicted persons. Research thus shows that the perception of dangerousness of the job is positively associated with job dissatisfaction among prison officers (Cullen et al., 1985) and negatively associated with job satisfaction of jail staff (Lambert and Paoline, 2008) and prison staff (Jiang et al., 2018).

In addition to the perceived dangerousness of the job, the prison officers’ job satisfaction can also be affected by job stress. Researchers describe job stress as psychological stress originating from a person’s job or work environment (Castle, 2008; Paoline and Lambert, 2012). Due to its impact on people, it is considered an important factor in understanding employee behavior in organizational environments. Research thus shows that high levels of job stress are associated with low levels of job satisfaction (Lambert et al., 2007; Hogan et al., 2009; Frank et al., 2019). Individuals who experience lower levels of job stress are more satisfied with their jobs, which applies to both jail officers (Castle, 2008) and private prison staff (Hogan et al., 2009). Based on this, the following hypotheses were formulated:

*H2a*: Organizational justice is positively associated with prison officers’ job satisfaction.

*H2b*: Dangerousness of the job is negatively associated with prison officers’ job satisfaction.

*H2c*: Job stress is negatively associated with prison officers’ job satisfaction.

In past research, the aforementioned factors that influence job satisfaction have also been associated with job involvement. Prison officers or other prison staff who perceive a higher level of organizational justice feel more involved in their work than those that perceive lower levels of organizational justice (Lambert et al., 2019). The danger of the job can also affect the job involvement of prison officers. For example, Lambert et al. (2014, 2018), and Lambert and Paoline (2012) found that if prison staff perceive their jobs as dangerous, that decreases their job involvement. Based on the results of previous research, a negative correlation between job involvement and job stress can also be assumed (Griffin et al., 2010; Paoline and Lambert, 2012; Lambert et al., 2017); staff with high job involvement experience less stress at work than staff with low job involvement. Based on this, the following hypotheses were formulated:

*H3a*: Organizational justice is positively associated with prison officers’ job involvement.

*H3b*: Dangerousness of the job is negatively associated with prison officers’ job involvement.

*H3c*: Job stress is negatively associated with prison officers’ job involvement.

**Figure 1:**
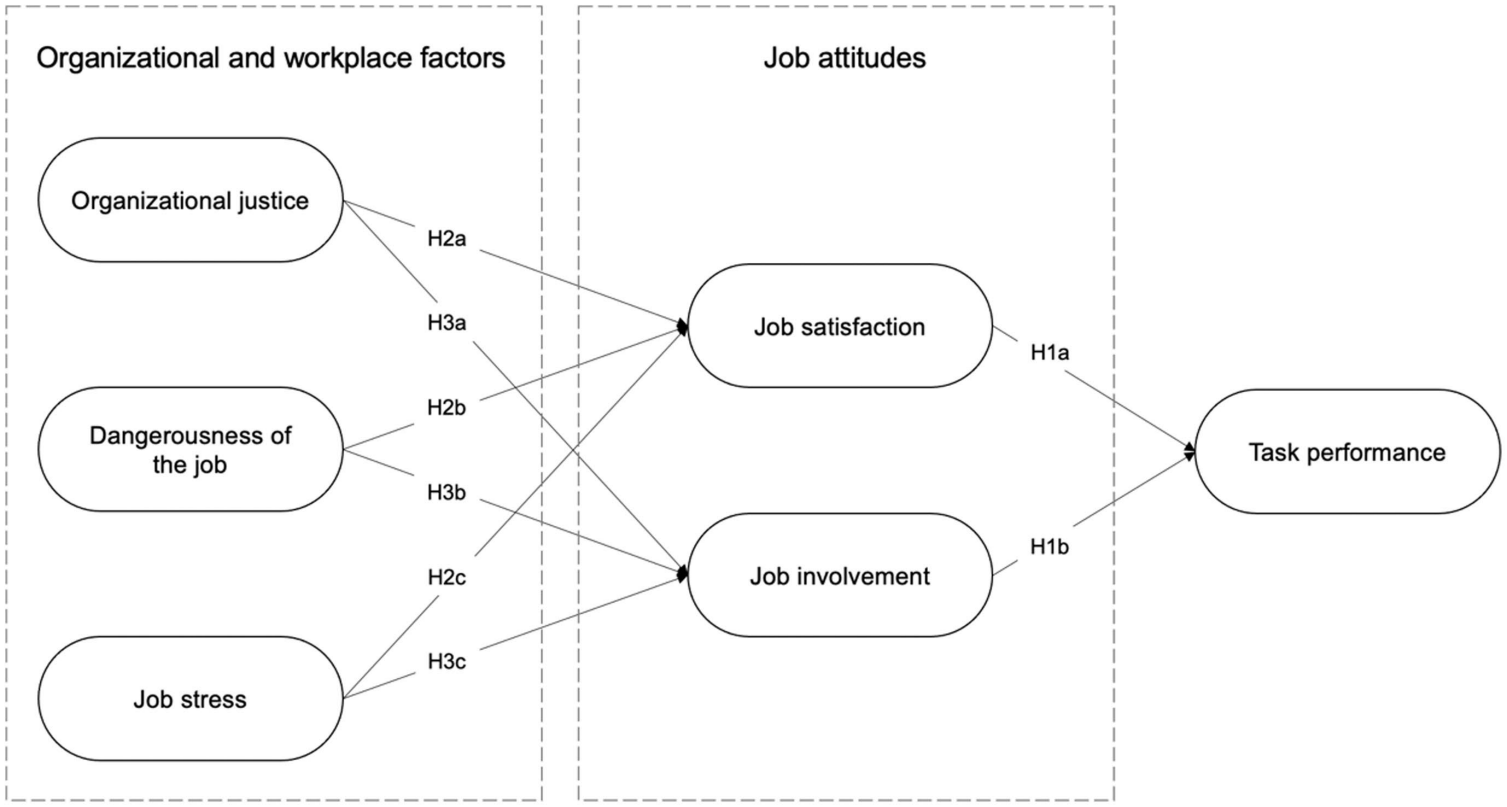
Research model with hypotheses.

For a better illustration, the hypotheses presented above are also shown in the following visualization of the research model used in this study (Figure 1).

## 5. Methods

### 5.1. Study background: the review of the Slovenian prison system

Prisons in Slovenia are internal organizational units of the Prison Administration of the Republic of Slovenia, which is a body of the Ministry of Justice. The administration’s vision focuses on the effective enforcement of sanctions and the establishment of an excellent prison system with highly qualified, motivated, and goal-oriented staff. In this regard, it works toward providing support for convicted persons and enabling their participation in social reintegration programs as one of the purposes of a prison sentence. The administration’s main mission is to ensure the enforcement of criminal sanctions, detention, prison sentence, alternate prison sentence, and the educational measure of placing a minor into a correctional home. It strives for prisons that are safe for the society, the staff, and the prisoners (Prison Administration of the Republic of Slovenia, 2016).

The administration is in charge of six prisons (in Celje, Ljubljana, Ig, Dob, Koper, and Maribor), a correctional home in Radeče, and the closed, open, and semi-open units within individual prisons (Prison Administration of the Republic of Slovenia, 2021). Even though all the prisons and the correctional home perform the same function, they cannot be considered in the same way, at least not in all respects. For example, the Radeče Correctional Home holds juveniles from age 14 onward. It carries out the educational measure of placing a minor into a correctional home, using highly qualified staff (psychologists, sociologists, etc.) to help the juveniles and prevent them from committing further criminal offenses. The Koper, Ljubljana, Dob and Maribor prisons hold men over the age of 18. They comprise various additional units across the country, some of which also have open and semi-open units, and hold convicted persons and detainees. The prison in Ig also houses women over the age of 18, while the Celje Juvenile and Adult Prison also holds convicted minors (Prison Administration of the Republic of Slovenia, 2021). In Slovenia, convicted persons are thus classified by age (juvenile and adult), gender (men and women), regime (open, closed, and semi-open units), and sentence.

At the time this study was conducted, the aforementioned facilities employed 545 prison officers: 489 men and 56 women (Prison Administration of the Republic of Slovenia, 2021). It should be noted that the prison officers’ role is not only to protect individuals and the facilities, but also to enforce the prison’s house order, escort inmates to court and health institutions, and supervise visits and individual prison areas via security cameras (Zakon o izvrševanju kazenskih sankcij (ZIKS-1), 2006; Prison Administration of the Republic of Slovenia, 2021).

### 5.2. Questionnaire development

The present study included a survey that evaluated the prison officers’ self-reported task performance and examined their job attitudes and perceptions of various organizational and work-related factors. Based on the information gathered, the study then examined the impact of the prison officers’ job attitudes on their task performance and the correlations between other factors and their job attitudes. The constructs included in the questionnaire were formulated and adapted to the research topic based on the review of previous research in this area (see Table 1). Every construct comprised several questionnaire items (see Table 2) provided in the form of statements and analyzed on a 5-point Likert scale (1 – completely disagree, 5 – completely agree).

**Table 1:** Questionnaire design.

Constructs	Items	Sources
Task performance	5	Williams and Anderson (1991)
Job involvement	3	Kanungo (1982)
Job satisfaction	3	Brayfield and Rothe (1951)
Organizational justice	3	Ambrose and Schminke (2009)
Job stress	3	Cullen et al. (1985)
Dangerousness of the job	3	Cullen et al. (1985)

**Table 2:** Rotated factor matrix.

	Factor					
	1	2	3	4	5	6
*Task performance*						
I adequately complete assigned job duties.	0.848					
I meet formal job performance requirements.	0.868					
I fulfill the responsibilities specified in my job description.	0.879					
I engage in activities that will directly affect my job performance evaluation.	0.629					
I perform job tasks that are expected of me.	0.847					
*Job stress*						
A lot of times, my work makes me very frustrated or angry.					0.699	
Most of the time, I am under a lot of pressure at my work.					0.812	
I often feel tense or uptight at my work.					0.852	
*Job satisfaction*						
I am enthusiastic about my job.		0.843				
I find real enjoyment in my job.		0.865				
I am satisfied with my job.		0.799				
*Organizational justice*						
Overall, I am treated fairly by the organization I work for.				0.831		
In general, I can count on the organization I work for to be fair.				0.927		
Most of my colleagues - prison officers would say that they are treated fairly by the organization they work for.				0.711		
*Dangerousness of the job*						
A prison officers’ job is dangerous.			0.872			
A prison officers’ job is more dangerous than other jobs.			0.785			
There is a risk of injury working as a prison officer.			0.814			
*Job involvement*						
My job is a big part of who I am.						0.553
I am very much involved personally in my job.						0.791
I have very strong ties with my job, which would be very difficult to break.						0.752

Factor extraction: principal axis factoring rotation: varimax with Kaiser normalization.

The questionnaire items were initially written in English, after which they were sent to three separate translators, who translated them into Slovenian. Based on this, a uniform Slovenian version of the questionnaire was created and sent to three reviewers to ensure clarity. After obtaining their feedback and having the suitability of the questionnaire confirmed by the Prison Administration of the Republic of Slovenia, the final version of the questionnaire was created and sent to the target population.

### 5.3. Data collection

Approval to conduct the survey was obtained from the Prison Administration of the Republic of Slovenia in March 2021. The target population included all prison officers in Slovenia. As such, all prison officers at the six prisons mentioned above (i.e., the Dob, Ig, Ljubljana, Maribor, and Koper prisons, and the Celje Juvenile and Adult Prison) and the Radeče Correctional Home were invited to participate in the survey.

The questionnaire was available in paper and online versions. Respondents could choose between the two, but they were only allowed to complete the questionnaire once (either on paper or online). An invitation to participate in the survey and a link to the online questionnaire were disseminated through the administration’s internal communication channels, and the paper questionnaires were sent to every institution by mail. The invitation explained that participation in the survey was voluntary and anonymous.

The survey was conducted between 19 March and 28 May 2021. During that time, the Prison Administration of the Republic of Slovenia also sent out two reminders to all prison officers (the first a week after the initial invitation and the second two weeks after that). The Ethics Committee of the Faculty of Criminal Justice and Security has affirmed that the research conducted adhered to the University of Maribor’s code of ethics. This confirmation is documented in Report No. 0506-2023.

### 5.4. Sample

A total of 201 prison officers participated in the survey. The sample thus covered 36.8% of all prison officers employed within the Slovenian prison system. Most respondents filled out the paper version of the questionnaire (59.2%), while others participated in an online survey. Due to the mixed approach to data collection, we tested these two groups for differences in their responses. Results of the independent sample t-test showed no statistically significant differences, except for one indicator of one construct. Hence, the two groups were combined and analyzed as one sample. Table 3 shows the demographic characteristics of the sample.

**Table 3:** Sample characteristics.

	*n*	%
*Gender*		
Male	170	84.6
Female	30	14.9
Not provided	1	0.5
*Age*		
21–33 years	27	13.4
34–46 years	119	59.2
47–60 years	48	23,9
Not provided	7	3,5
*Years of working experience*		
Less than 10 years	98	48.8
11–20 years	57	28.4
More than 21 years	46	22.9
*Years of employment at the current institution*		
Less than 10 years	106	52.7
11–20 years	54	26.9
More than 21 years	41	20.4
*Management position*		
Yes	70	34.8
No	128	63.7
Missing values	3	1.5

The respondents included 170 men (84.6%) and 30 women (14.9%), while one respondent did not indicate his or her gender. Most respondents (59.2%) were between 34 and 46 years old, and the fewest belonged to the age group between 21 and 33 years (13.4%). The average respondent age was 42 years; the youngest respondent was 21 and the oldest was 60. On average the respondents had worked as prison officers for nearly 13 years, and had been with their current institution for 12 years.

### 5.5. Instrument validation

Before answering the research questions, we tested the questionnaire for validity and reliability. All statistical analyses in this study were conducted with IBM Statistics SPSS v28. To test the validity, we conducted an exploratory factor analysis (Prinicpal Axis Factoring) with an orthogonal rotation (Varimax), which extracted six theoretically based factors. With those factors, 72.04% variance can be explained. The Kaiser-Meyer-Olkin (KMO) measure of sampling adequacy is 0.812, which indicates that these data are suitable for conducting factor analysis. The Bartlett’s test of sphericity was statistically significant (*χ*^2^ = 2787.319, *df* = 190, *p* < 0,001). The rotated factor matrix with factor loadings is presented in Table 2. Because of transparency, only factor weights greater than 0.3 are presented.

The questionnaire reliability was measured with Cronbach Alpha (CA) coefficient. The values of Cronbach’s alpha (Table 4) range between 0.781 and 0.934.

**Table 4:** Cronbach’s alpha.

Constructs	Items	Cronbach’s alpha
Task performance	5	0.913
Job stress	3	0.843
Job satisfaction	3	0.934
Organizational justice	3	0.878
Dangerousness of the job	3	0.898
Job involvement	3	0.781

## 6. Results

### 6.1. Descriptive statistics

To answer the first research question, the descriptive statistics were first calculated. Table 5 presents the results for the constructs and individual items in the form of arithmetic means (*M*), standard deviations (*SD*), medians (*Mdn*), and modes (*Mo*).

**Table 5:** Descriptive statistics.

Constructs and items	*M*	*SD*	*Mdn*	*Mo*
Task performance	4.66	0.57	5.00	5
I adequately complete assigned job duties.	4.71	0.61	5.00	5
I meet formal job performance requirements.	4.62	0.67	5.00	5
I fulfill the responsibilities specified in my job description.	4.70	0.61	5.00	5
I engage in activities that will directly affect my job performance evaluation.	4.51	0.82	5.00	5
I perform job tasks that are expected of me.	4.78	0.60	5.00	5
Job satisfaction	3.84	0.95	4.00	5
I am enthusiastic about my job.	3.73	1.06	4.00	4
I find real enjoyment in my job.	3.76	1.03	4.00	4
I am satisfied with my job.	4.03	0.93	4.00	4
Job involvement	3.20	0.95	3.00	3
My job is a big part of who I am.	3.54	1.09	4.00	4
I am very much involved personally in my job.	3.25	1.15	3.00	3
I have very strong ties with my job, which would be very difficult to break.	2.81	1.18	3.00	3
Organizational justice	3.27	0.95	4.00	5
Overall, I am treated fairly by the organization I work for.	3.50	1.30	4.00	4
In general, I can count on the organization I work for to be fair.	3.44	1.19	4.00	4
Most of my colleagues - prison officers would say that they are treated fairly by the organization they work for.	2.88	1.03	3.00	3
Job stress	2.41	0.96	2.33	2
A lot of times, my work makes me very frustrated or angry.	2.26	1.01	2.00	2
Most of the time, I am under a lot of pressure at my work.	2.81	1.24	3.00	2
I often feel tense or uptight at my work.	2.16	1.02	2.00	2
Dangerousness of the job	4.17	0.91	4.33	5
A prison officers’ job is dangerous.	4.13	0.99	4.00	5
A prison officers’ job is more dangerous than other jobs.	4.17	1.02	4.00	5
There is a risk of injury working as a prison officer.	4.20	0.99	5.00	5

Based on the descriptive statistics, it can be concluded that prison officers evaluated their own task performance as very high (*M* = 4.66), which means they are very confident about the quality of the work they perform and evaluate the efforts they invest in fulfilling their job requirements and responsibilities extremely positively. Specifically, they assigned the highest ratings to their performance of expected job tasks (*M* = 4.78) and the lowest (although still very positive) to their engagement in activities that directly affect their job performance evaluation (*M* = 4.51).

Moreover, the results show that the prison officers are not dissatisfied with their job (*M* = 3.84). This means they mostly enjoy what they do. This is also confirmed by the median (4) and the mode (5) for this construct. The respondents agreed the most with the statement that they were satisfied with their job (*M* = 4.03), whereas they agreed the least with the statement that they were enthusiastic about their job (*M* = 3.73).

The results for the job involvement construct were predominantly neutral (*M* = 3.21). The respondents agreed the most with the statement that their job was a great part of who they were (*M* = 3.54), whereas they agreed the least with the statement that they had very strong ties with their job (*M* = 2.81).

Most respondents also believed the organization they work for is fair (*M* = 3.27). The respondents were the least convinced that their colleagues would say they are treated fairly by the organization they work for (*M* = 2.88). However, they agreed most strongly with the statement that they are personally treated fairly by their organization (*M* = 3.5).

There is not much job stress present among the prison officers (*M* = 2.41), which means they do not feel tense or uptight and are not under a lot of pressure at work. The values of all indicators are below the mean (3). The respondents agreed the most with the statement that they were under pressure at work (*M* = 2.81) and the least with the statement that they felt uptight at work (*M* = 2.16).

Despite these positive views and feelings, the respondents nonetheless perceive their job to be dangerous (*M* = 4.17), which means they are aware of the risks associated with their work. They agreed there is a risk of injury at their workplace (*M* = 4.2), and that a prison officer’s job is dangerous (*M* = 4.13).

### 6.2. Research model testing

Three multiple linear regression models were tested to answer the second and third research questions. This statistical method allowed us to assess how were multiple independent variables associated with the dependent variables (i.e., task performance, job satisfaction, job involvement), providing insights into their individual and combined contributions. To test discriminant validity, Pearson correlations were calculated to check the inter-construct correlations (results are presented in Table 6). The test results showed statistically significant correlations between task performance and (1) job satisfaction (*r* = 0.358; *p* < 0.01), (2) dangerousness of the job (*r* = 0.377; *p* < 0.01) and (3) job involvement (*r* = 0.204; *p* < 0.01). In addition, statistically significant correlations were found between job satisfaction and (1) job stress (*r* = −0.314; *p* < 0.01), (2) organizational justice (*r* = 0.378; *p* < 0.01) and (3) job involvement (*r* = 0.328; *p* < 0.01). In addition to its already mentioned statistically significant correlation with task performance and job satisfaction, the job involvement construct also shows statistically significant correlation with (1) organizational justice (*r* = 0.281; *p* < 0.01) and (2) dangerousness of the job (*r* = 0.314; *p* < 0.01). All the correlation coefficients are below the recommended threshold (*r* < 0.7), which confirms the appropriate discriminant validity of the model. In addition, other linear regression assumptions were also carefully taken into account (e.g., multicollinearity, homoscedasticity and normal distribution of residuals).

**Table 6:** Pearson correlation coefficient.

	1	2	3	4	5	6
1. Task performance	1					
2. Job stress	−0.125	1				
3. Job satisfaction	0.358^**^	−0.314^**^	1			
4. Organizational justice	0.138	−0.123	0.378^**^	1		
5. Dangerousness of the job	0.377^**^	0.167^*^	0.128	0.058	1	
6. Job involvement	0.204^**^	0.128	0.328^**^	0.281^**^	0.314^**^	1

*p < 0.05; **p < 0.01.

**Figure 2:**
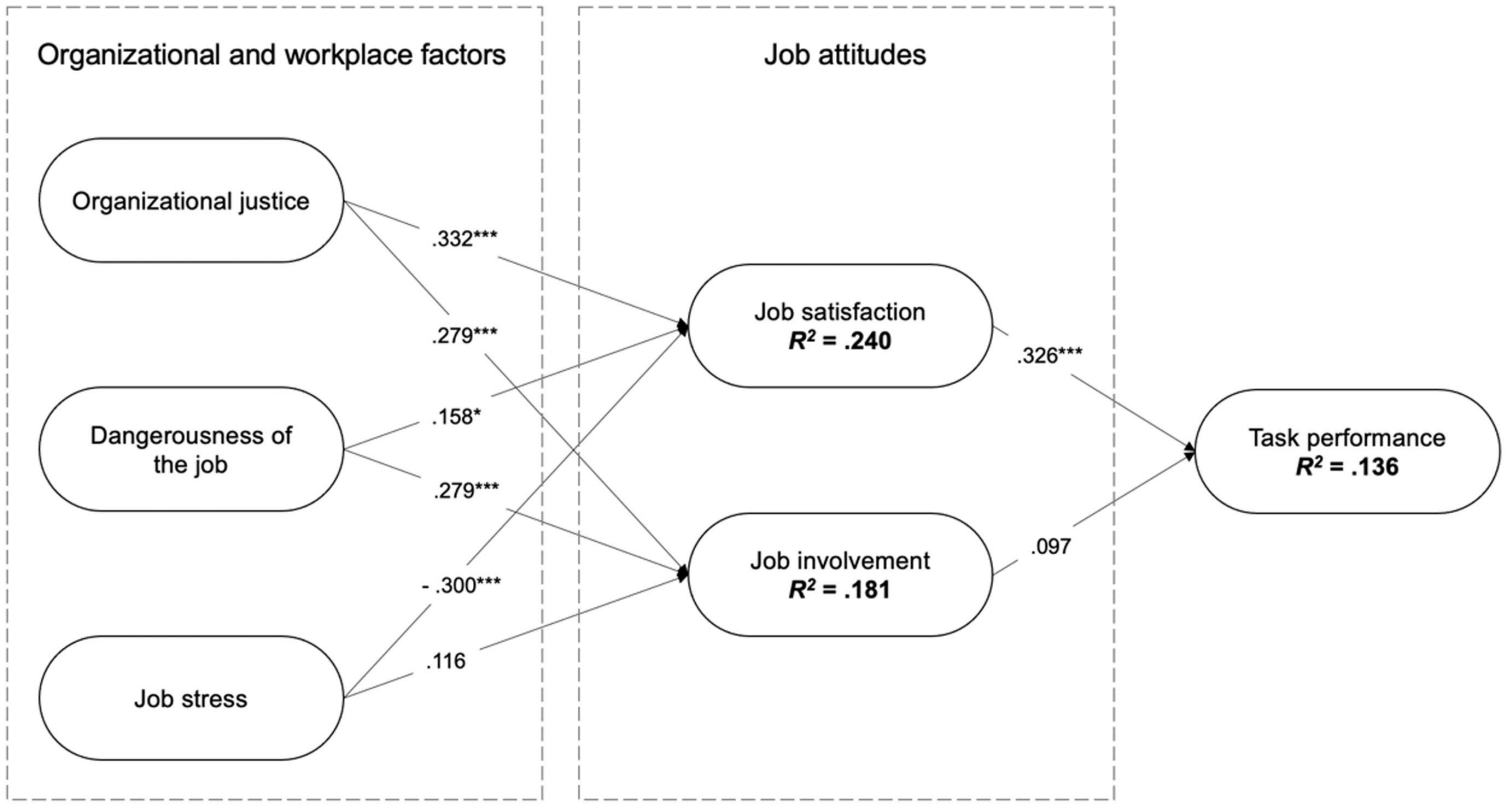
Model testing results.

Three linear regression analyses were conducted to test the theoretical research model and hypotheses. The results of multiple regression analyses (Table 7; Figure 2) are presented.

**Table 7:** Multiple regression analyses results.

Model	*β*	*t*	Sig.	S.E.	VIF	*F*	*R* ^2^
*Model 1: task performance*							
Job satisfaction	0.326	4.661	0.000	0.42	1.121	15.641***	13.6%
Job involvement	0.097	1.389	0.166	0.42	1.121		
Model 2: job satisfaction							
Organizational justice	0.332	5.284	0.000	0.056	1.022	20.707***	24.0%
Dangerousness of the job	0.158	2.506	0.013	0.066	1.035		
Job stress	−0.300	−4.714	0.000	0.063	1.048		
Model 3: job involvement							
Organizational justice	0.279	4.281	0.000	0.059	1.022	14.507***	18.1%
Dangerousness of the job	0.279	4.251	0.000	0.068	1.035		
Job stress	0.116	1.753	0.081	0.066	1.048		

*p < 0.05; **p < 0.01; ***p < 0.001.

All regression models are statistically significant (Table 7; *p* < 0.001). Model 1 analyzed how job satisfaction and job involvement are associated with task performance. This can explain 13.6% of the variance in the reported task performance. Model 2 analyzed how organizational justice, dangerousness of the job, and job stress are associated with job satisfaction. This can explain 32.6% of the variance in the reported job satisfaction. Model 3 analyzed how organizational justice, dangerousness of the job, and job stress are associated with job involvement. This can explain 18.1% of the variance in the reported job involvement.

As seen in Figure 2, the results of the first regression model show that of the two factors related to job attitudes, the prison officers’ task performance is associated the most with their job satisfaction, whereas no association was confirmed with the second factor – that is, job involvement. It can be concluded that improved job satisfaction can also improve the prison officers’ task performance (*β* = 0.326; *p* < 0.001). Based on these results, hypothesis H1a (job satisfaction is positively associated with prison officers’ task performance) can be confirmed, unlike hypothesis H1b (job involvement is positively associated with prison officers’ task performance), which cannot be confirmed.

To better understand the circumstances that may also indirectly prove relevant to the prison officers’ job performance, Models 2 and 3 were used to establish how organizational and work-related factors were associated with job attitudes. The second regression model showed that job satisfaction is associated the most with perceived organizational justice (*β* = 0.332; *p* < 0.001), followed by job stress (*β* = −0.300; *p* < 0.001), and the least with the dangerousness of the job (*β* = 0.158; *p* < 0.05). Accordingly, hypotheses H2a (organizational justice is positively associated with prison officers ‘job satisfaction) and H2c (job stress is negatively associated with prison officers’ job satisfaction) can be confirmed, which cannot be claimed for hypothesis H2b (dangerousness of the job is negatively associated with prison officers’ job satisfaction).

The third regression model established that job involvement is associated with organizational justice (*β* = 0.279; *p* < 0.001) and the dangerousness of the job (*β* = 0.279; *p* < 0.001). Based on this, hypothesis H3a (organizational justice is positively associated with prison officers’ job involvement) was confirmed, whereas hypotheses H3b (dangerousness of the job is negatively associated with prison officers’ job involvement) and H3c (job stress is negatively associated with prison officers’ job involvement) were not. It can be concluded that improved organizational justice also increases job satisfaction and job involvement, and the same association with job attitudes can also be established for the dangerousness of the job. In contrast, increased job stress reduces job satisfaction.

## 7. Discussion

The concept of employee job performance has been the subject of organizational studies for years, as its understanding and management contribute significantly to organizational effectiveness. Despite such research is common in other professions the results cannot be generalized for the prison system. To some extent, there are comparable studies in other security professions, namely police; however, prisons are a closed system, so research like this is needed. It is also impossible to generalize the results of related research from other countries in the US or Western Europe because the cultural context also plays an important role. Considering the fact that such research is lacking in the post-socialist countries of Central and Southeastern Europe, our study presents a valuable contribution to the literature.

This article focused on examining the task performance of prison officers within the Slovenian prison system and establishing how job attitudes impact task performance. It also examined which organizational and work-related factors can be indirectly associated with task performance via their impacts on job attitudes.

The study’s findings indicate extremely high self-reported task performance of prison officers. Even though the reasons for the high-rated task performance could be ascribed to reporting errors [e.g., the overconfidence effect or social desirability; (Holzbach, 1978)], the results may also reflect respondents’ sincere beliefs about fulfilling all their official job tasks and requirements well and with high quality. This may also stem from the fact that prison officers working with inmates in highly dangerous conditions must always be well prepared and regularly participate in various education and training activities. For example, based on Slovenian regulation, prison officers must carry out at least 2 h of training per month to sustain and upgrade their work skills. In addition to the expertise obtained through their formal education, they must also have the necessary informal skills (i.e., social and communication) to carry out all their duties.

In discussing the reality of the established high task performance of prison officers, the context of the conducted research must also be taken into account. In Slovenia, the penal policy and thus the system of enforcing criminal sanctions became much stricter in the period after gaining independence. Although Slovenia has a relatively low incarceration rate and is comparable to Scandinavian countries, it has faced an increase in the prison population in the past decades. The Slovenian prison system strengthened its security component and slightly reduced its rehabilitation role, which became of secondary importance to the goal of maintaining order and security in prisons. In line with the trends in the prison population, the number of judicial police officers has increased slightly, and the penal profession has become more professionalized and directed toward employee training. Despite opening new organizational units and a slight increase in personnel capacity, the system still faces overcrowding, high workload, and understaffing. Due to the lack of professional staff, judicial officers began to take over some treatment tasks (Hacin and Meško, 2020). Taking into account the increased workload and the complexity, variety, and danger of the tasks performed by prison officers, we can understand why they see and value their contribution to the success of prison operations and evaluate their work results with such optimism and confidence.

In testing the hypotheses, we established that job satisfaction, as an aspect of job attitudes, is positively associated with task performance. This means that increased job satisfaction also increases the prison officers’ task performance, which is consistent with the findings of other researchers (e.g., Vandenabeele, 2009; Frank et al., 2019). In turn, we did not find job involvement, as another aspect of job attitudes, to be associated with the self-evaluated task performance of the prison officers. Even though this correlation has been established by some former studies (e.g., Johari and Yahya, 2016; Hermawati and Mas, 2017; Thevanes and Dirojan, 2018), the findings of research to date have not been entirely consistent. For example, in a survey conducted among the correctional staff at an American prison, Lambert et al. (2008) did not confirm these correlations, highlighting the possibility of indirect influences and moderator variables linking job involvement with job performance (e.g., organizational commitment).

Our study showed that perceived organizational justice is positively associated with prison officers’ job satisfaction. This means that if they believe they are treated fairly by their organization, this positively affects their satisfaction with work. This finding can be substantiated by the results of previous studies, in which Lambert et al. (2018) and Frank et al. (2019) also established the same correlation. In addition, we determined that the perceived dangerousness of the job affects job satisfaction. Authors, such as Castle (2008), Jiang et al. (2018), and Qureshi et al. (2019), have already directed attention to this issue in their research, but the results vary; in some studies, the dangerousness of the job was found to affect job satisfaction, whereas in others this correlation was not confirmed (e.g., Castle, 2008; Lambert and Hogan, 2009). The results of our regression analysis also show a negative correlation between job stress and job satisfaction, which means that increased stress reduces job satisfaction. This has also been confirmed by other studies conducted in the prison context (e.g., Lambert and Paoline, 2012; Frank et al., 2019).

Moreover, job involvement is also associated with organizational justice, which has been relatively consistently confirmed by previous research (e.g., Lambert et al., 2019; Qureshi et al., 2019), and with the dangerousness of the job. However, the findings of previous studies have been more inconsistent on the latter. For instance, Cullen et al. (1985) and Lambert and Paoline (2012) established no correlation between the dangerousness of the job and job involvement, whereas Lambert et al. (2013, 2018) reported a negative correlation. Furthermore, our study demonstrated that job stress does not affect job involvement, whereas this correlation has previously been confirmed by some other studies conducted among prison staff (Griffin et al., 2010; Paoline and Lambert, 2012; Lambert et al., 2017).

In essence, our research showed that job involvement and job satisfaction, which were reported as high and neutral respectively, are affected by both perceived organizational justice and the danger of the job. Perceived dangerousness of the job was rated high, which indicates that the prison officers are aware of the risks and dangers associated with their profession. They rated organizational justice very positively, reporting that they can count on the organization they work for to be fair, but they were more critical with regard to how their colleagues would evaluate the same fairness. Even though prison officers do not perceive their job as stressful, stress nonetheless affects their job satisfaction, but it does not impact their job involvement.

These results have an important added value, especially in understanding the implications of job stress and dangerousness. Although stress at work affects prison officers’ job attitudes to some extent, it does not reduce their job involvement. Such a conclusion could be ascribed to the fact that the respondents are exposed to stressful situations and job stress daily. We can assume that stress is something that prison officers have gotten used to throughout their careers (on average, the respondents have worked in this profession for nearly 13 years and had already been employed at a single institution for an average of 12 years), and thus have already internalized it. The fact that they had been working at the same place and among the same or similar staff for so long, and thus in a familiar environment, could be the reason why they do not perceive their work to be so stressful that it would negatively affect their job involvement. However, job stress does have a negative effect on their job satisfaction. In conclusion, job stress does not make the prison officers any more or less involved with their work, but it does affect their satisfaction with it. This makes sense, as if employees experience great stress at work, this can also have negative psychological, physical, and behavioral effects, which may also impact overall satisfaction with the work they perform (Frank et al., 2019).

Furthermore, we found that the dangerousness of the job does not cause a reduction in job satisfaction or job involvement. Thus, the awareness that their work is dangerous does not constitute a disturbing factor for prison officers, just the opposite. Therefore, it is not an indicator of any decrease in prison officers’ job attitudes but in fact, contributes to greater job satisfaction and involvement. A possible reason for this could be the fact that the desired characteristics of prison officers include high physical fitness and other skills required to deal with dangerous situations (Meško et al., 2004), which is why these dangers do not decrease their well-being at work, but allow them to apply the skills they have. However, this finding disagrees with the results of previous research, for which there may be various reasons. In explaining the diverse findings of previous studies, the potential effects of different types of organizations must not be ignored. Specifically, while the impact of the dangerousness of the job has often been examined in a prison setting, the related studies were conducted in various environments, such as jails, high-security prisons, and open-type prisons, which house different groups of inmates and require different levels of security (Castle, 2008; Lambert and Paoline, 2012; Jiang et al., 2018), and this could affect the staff’s perception of job dangerousness. These studies also took place in different countries with different prison systems, which makes it more difficult to compare the results. Ultimately, the reason for the inconsistent findings in the literature may thus be the differences in the organizational and social climate of the prisons studied.

To sum up, higher job performance was found among those prison officers who reported being satisfied with their work, which is why maintaining high job satisfaction among prison officers is vital for effective and successful performance of their basic tasks. However, because their job satisfaction is affected by perceived (1) organizational justice, (2) dangerousness of the job, and (3) job stress, it can be concluded that to ensure high employee task performance, it is also necessary to manage these factors through suitable strategic, managerial and organizational measures. In addition, this study showed that as an organizational factor, organizational justice is more significantly associated with job satisfaction and job involvement than the other two work-related factors, which provides more detailed insight into which aspects should be given priority in managing employee job performance.

Although our research did not yield evidence supporting a statistically significant association between job involvement and task performance, the potential importance of this aspect of job-related attitudes should not be disregarded. By taking into account the extant literature and preliminary studies, it becomes evident that job involvement among employees signifies elevated levels of employee motivation, leading to endeavors to attain superior performance and enhanced productivity. Heightened job involvement can foster increased employee engagement in work processes, proactive problem-solving, willingness to assist others, and ultimately higher-quality outcomes. The relationship of job involvement with task performance may also be indirect, with job satisfaction mediating the relationship, highlighting the imperative role of job involvement as a driver of employee performance.

The present study is an important addition to the existing body of literature because previous studies in a prison environment only rarely focused on task performance as a specific dimension of job performance and associated job attitudes with job performance. Moreover, this is the first study in the Slovenian prison environment examining the job performance of prison officers. Therefore, it constitutes an original work that can serve as a starting point for further research in this area. Last but not least, the results of this study and its theoretical research model form the basis for other researchers’ investigations of prison officers’ workplace behavior.

The findings of this study also have practical implications. First, they are relevant for prison officers. The fact is that feedback is key for the development and progress of employees and the improvement of their work. Second, the results also give senior officers, prison management, and the Prison Administration insight into the current situation. They can use these findings to better understand the prison officers’ behavior, identify possible improvements, and address the weaknesses. Third, the findings can also prove useful for other stakeholders in law enforcement and security organizations, where staff work under similarly dangerous conditions.

### 7.1. Limitations and future work

The main limitations of this study are related to the process of data collection. First, at the time of the study, the COVID-19 pandemic and related measures were declared in Slovenia. For this reason, the researchers were unable to physically access the respondents. The invitations and questionnaires were thus delivered to them remotely. Second, a further limitation is related to the self-evaluation of job performance, which requires the results to be interpreted with caution. Although most studies have evaluated job performance based on employee self-reports or self-ratings, self-evaluations can be problematic in terms of validity and reliability of the results due to potential measurement errors, such as biases and providing socially accepted answers (Holzbach, 1978). However, some authors (e.g., Kock, 2017) consider self-perceptions to be a more appropriate approach than official supervisor evaluations, because a different problem can emerge in this case: the absence of an objective, critical perspective, resulting from the desire to stay in good relations with the employees. In addition, self-evaluations allow for greater anonymity of answers than the official performance evaluations produced by supervisors. Third, the study was conducted only in one European country; thus, the results may not be applicable in other countries due to the differences in prison, criminal justice, and political systems.

Even though the topics of organizational and job performance have become increasingly popular over the years, especially at a time of constantly changing work conditions and business circumstances, studies addressing these aspects continue to be quite rare, especially in law enforcement and in Slovenia. Moreover, most previous studies of this type focused exclusively on individual job performance dimensions, such as OCB or CWB. In contrast, more comprehensive studies that would simultaneously examine multiple dimensions remain to be conducted.

Further research would benefit from expanding the range of possible factors affecting job performance and job attitudes. The study presented in this article explained only 13.6% of the variance in task performance, which means that a significant share of the variance remains unexplained or could be explained with other variables not included in this study. In addition, the study explained only 24% of the variance in job satisfaction and only 18% of the variance in job involvement. To better understand the factors influencing prison officers’ job attitudes, it is thus key, to expand the range of factors studied and examine, for instance, how job burnout, work conditions, or interpersonal relations affect job satisfaction and job involvement.

To better understand the role and importance of job involvement, future studies could also explore the impact of job involvement on job satisfaction, job stress, and organizational commitment as suggested by Paoline and Lambert (2012), or examine job involvement through multiple dimensions (e.g., involvement in the work process or involvement in working with inmates).

Ultimately, the literature would benefit from enlarging the pool of comparative studies, such as different prison types and regimes comparisons at both the national level and beyond. Cooperation in studying work conditions at prisons, the prison officers’ work, and their well-being at work would also prove helpful in planning systemic improvements and identifying best practices. Based on the research model presented in this article, it would also make sense to conduct comparative studies with comparable law enforcement and security organizations (e.g., a police organization) to identify potential similarities and differences regarding the factors affecting the job performance of security staff.

## 8. Conclusion

The prison officers’ quality of work has many important implications for enforcing order and safety in prisons. By understanding the deficiencies in the staff’s current productivity and addressing factors that affect their job performance, it is possible to contribute to the more successful fulfillment of organizational objectives. This study showed that the prison officers’ task performance is heavily influenced by their job satisfaction, which in turn depends on perceived organizational justice, dangerousness of the job, and job stress. Therefore, the prison and prison system management must constantly and actively strive to maintain a high level of employee well-being by promoting open communication, good relations, mutual respect, and fair treatment of employees. At the same time, prison officers’ job performance should not be taken for granted. Instead, it is necessary to continuously evaluate and monitor it, and study its causes and effects. Only by consistently studying the prison environment and the prison officers’ well-being can we establish proper understanding and evidence-based improvements at an individual prison and thus support the entire prison system in achieving its vision and mission.

## Data availability statement

The datasets presented in this study can be found in online repositories. The names of the repository/repositories and accession number(s) can be found at: Mendeley Data: https://data.mendeley.com/datasets/ps2d37n45h.

## Ethics statement

The studies involving humans were approved by the Ethics Commission of the Faculty of Criminal Justice and Security University of Maribor. The studies were conducted in accordance with the local legislation and institutional requirements. The participants provided their written informed consent to participate in this study.

## Author contributions

KP, KPM, and AM contributed to conception and design of the study. KP, KPM, and BL collected data and organized the database. KP and KPM performed the statistical analysis and wrote the first draft of the manuscript. All authors contributed to manuscript revision, read, and approved the submitted version.

## Conflict of interest

The authors declare that the research was conducted in the absence of any commercial or financial relationships that could be construed as a potential conflict of interest.

## Publisher’s note

All claims expressed in this article are solely those of the authors and do not necessarily represent those of their affiliated organizations, or those of the publisher, the editors and the reviewers. Any product that may be evaluated in this article, or claim that may be made by its manufacturer, is not guaranteed or endorsed by the publisher.
